# Using Technology, Bioinformatics and Health Informatics Approaches to Improve Learning Experiences in Optometry Education, Research and Practice

**DOI:** 10.3390/healthcare4040086

**Published:** 2016-11-15

**Authors:** Vivek K. Gupta, Veer B. Gupta

**Affiliations:** 1 Faculty of Medicine and Health Sciences, Macquarie University, Sydney 2109, Australia; 2 School of Medical Sciences, Edith Cowan University, Perth 6027, Australia; v.gupta@ecu.edu.au

**Keywords:** optometry students, orthoptics, mobile technology, collaborative learning, inclusive teaching

## Abstract

Rapid advances in ocular diagnostic approaches and emerging links of pathological changes in the eye with systemic disorders have widened the scope of optometry as the front line of eye health care. Expanding professional requirements stipulate that optometry students get a meticulous training in relevant information and communication technologies (ICT) and various bioinformatics and health informatics software to meet current and future challenges. Greater incorporation of ICT approaches in optometry education can facilitate increased student engagement in shared learning experiences and improve collaborative learning. This, in turn, will enable students to participate in and prepare for the complex real-world situations. A judicious use of ICTs by teachers in learning endeavors can help students develop innovative patterns of thinking to be a successful optometry professional. ICT-facilitated learning enables students and professionals to carry out their own research and take initiatives and thus shifts the equilibrium towards self-education. It is important that optometry and allied vision science schools adapt to the changing professional requirements with pedagogical evolution and react appropriately to provide the best educational experience for the students and teachers. This review aims to highlight the scope of ICT applications in optometry education and professional development drawing from similar experiences in other disciplines. Further, while enhanced use of ICT in optometry has the potential to create opportunities for transformative learning experiences, many schools use it merely to reinforce conventional teaching practices. Tremendous developments in ICT should allow educators to consider using ICT tools to enhance communication as well as providing a novel, richer, and more meaningful medium for the comprehensive knowledge construction in optometry and allied health disciplines.

## 1. Introduction

Widespread and judicious use of ICT (information and communication technologies), including multimedia technologies, are increasingly playing an important role in learning and teaching activities and facilitate a shift from externally managed to self-directed learning [1]. Use of ICT technologies in combination with various bioinformatics and health informatics tools forms the bedrock of health sciences as well as biomedical sciences education. A widely-used model is to combine digital technologies with face-to face teaching for best educational outcomes. Needless to mention that this approach is more flexible and has greater capability to keep students engaged and is regarded as an effective way of delivering educational material [2]. Optometry is a fast-growing profession that addresses diagnosing and treating visual problems, prescribing eyeglasses or contact lenses and managing diseases, injuries, and other disorders of the eyes [3]. More recently, the scope of practice for optometrists is widening with newer regulations in different countries and as such there is a greater need for students to learn examining and make appropriate recommendations not only for eyes but also for ocular manifestations of potential systemic issues [4,5].

With an increasing number of students from diverse backgrounds opting for optometry and allied vision science courses, schools need to address the changing educational and professional requirements. In this regard, expertise in ICT and bioinformatics and health informatics technologies can provide students and professionals access to enormous amounts of biomedical and clinical data. As an example, competence in various bioinformatics tools can help in data mining and analysis of proteomics and genomics public data bases. Insights into genetic susceptibility and biomarker changes is likely to improve diagnostic accuracy and help develop personalized treatment for patients [6]. Similarly, knowledge in health informatics software and approaches can impart optometrists an ability to analyze data from complex imaging software such as magnetic resonance imaging/ positron emission tomography (MRI/PET) scanning to study higher visual centers in the brain and examine retinal imaging data from optical coherence tomography (OCT), retinal hyperspectral imaging and OCT angiography [7,8,9]. Improved access and skills to analyze MRI/PET data and longitudinal ocular imaging and electrophysiology history will help optometrists and ophthalmologists to make informed decisions about managing the patients [10]. These skills will be greatly helpful to correctly diagnose and investigate ocular manifestations of neurological disorders such as Alzheimer’s disease, optic neuritis and multiple sclerosis [11]. In addition, ICTs can facilitate assessing and analyzing laboratory reports such as those related to diabetes, hypertension, cardiac and respiratory function, cholesterol levels and blood/urine analysis and help optometrists to make informed diagnosis, prescribe drugs and making suitable referrals for complex ophthalmic and systemic problems [12,13]. This knowledge is essential with rapid technological advancements and promise to tilt the educational focus in optometry from factual recall to development of self-learning and problem solving skills and investigative attitudes. This ability will also expand the scope of practice and enable optometrists to perform more informed diagnosis, treatment and referral of the patients.

Optimizing the use of ICTs is particularly more relevant in the optometry profession as optometrists around the globe are increasingly playing a greater role in ocular diagnostics and are in the front line of eye care by linking with the ophthalmologists and eye hospitals virtually. With the increase in ageing populations, the prevalence of eye diseases is increasing and most places are struggling to have enough doctors to see the patients. This is especially critical for rural places but even in cities patients are experiencing disproportionately long waiting times for doctors’ appointments. Optometrists taking eye photographs; performing OCTs; doing visual field tests, electroretinograms and visual evoked potentials; checking intraocular pressures; and managing patients in an ophthalmology led, but virtual team, is happening [14,15]. Optimizing the use of ICTs then means that online case management can be more easily translated. Further, competing demands from clinical, administrative and research sectors for optometry professionals and teaching faculty makes professional development often opportunistic rather than interest based and familiarity with a wider range of skills will facilitate interest based collaborative professional development [16]. This competence will also help diversify research skills of optometrists and enable them to explore frontier areas of vision sciences.

Wider implementation of ICTs in optometry education can face initial challenges from professionals who are already well established in their comfort zone [17,18]. However, the ones who have expertise in ICT use are eventually more likely to contribute to expanding the frontiers of the discipline, drawing knowledge from education, computer, optical, other health care and biomedical disciplines. Implementation hurdles can gradually be overcome with the support of professional bodies with a broad aim to explore the frontiers of optometry, orthoptics and ophthalmology education [19].

Most optometry and orthoptics training institutions are already using varying levels of ICT based teaching and learning practices, however sustainable use of technology and ability to electronically access and analyze data is still in its infancy and this is true for most of the health care professional studies. Presently, the question is not whether the course content such as lectures, seminars and other virtual learning experiences should or should not be offered to students, but rather how best these tools and mediums can be used to enrich the learning experiences. ICTs can also open up vast online learning options and can play an important role in telemedicine which has tremendous applications for remote areas. It also encompasses all electronic media for presentation, communication and circulation of learning material which can be used as an add-on to enhance conventional classroom teaching experiences or as blended learning. Another aspect is that greater emphasis on ICT technologies is more economical in the longer run as it enables institutions to divert resources to other aspects of optometry education such as more clinical and research oriented learning [20,21,22]. Greater use of ICT for online learning activities helps students and teachers to engage, independent of place and time, for better educational prospects. Briefly, while ICT approaches are being increasingly used in several disciplines, their usage in various health disciplines including in optometry and orthoptics is rather limited and needs to be stepped-up [23]. The present review discusses strategies, prospects and hurdles for the wider implementation of ICT mediated learning in optometry and gives an overview of the perception of students and teachers to these changes.

## 2. Methodology

Literature search: A thorough literature search was carried out to identify the articles related to information and communication technology in higher education in health sciences with particular focus on optometry, orthoptics and vision science education. Relevant key words such as optometry education, online learning, tertiary education, medical students, mobile technology in learning, inclusive education, bioinformatics and health education, health informatics, teachers, virtual learning were searched in Medline, Google scholar and Web of Science collections. Articles and books published in English language were only considered. Websites of several optometry and ophthalmology professional bodies, vision science funding organizations and universities offering optometry courses were searched.

## 3. ICT Categories with Potential Educational Value in Optometry

Digital education approaches and their impact can vary greatly depending on the type and context of technologies used for the learning activities. In optometry, this may include offline and online computer-based e-learning, bioinformatics, health informatics and virtual reality environments [24]. 

In offline learning, the educational activities are carried out through an individual computer, software and other digital equipment involving task specific techniques. Content delivery can be through data discs, memory drives etc. Local area network-based learning is also often used to access various e-books and scholarly papers through the library repositories. Such offline digital learning is generally considered equivalent and might be better than traditional paradigms in terms of student engagement, knowledge acquisition, skill development and student and teacher satisfaction [25]. This approach can be easily adapted in most educational and clinical environments in optometry and can be greatly beneficial if used concurrently with traditional learning systems. Various tools involving audio based technologies (voice tools), video based resources (iMovie), graphical resources such as adobe photoshop, coral draw, paint etc., can be used. Digital-learning is also increasingly being used for psychological and motor skills training in several health care training courses, to improve motor co-ordination skills and learning the correct ways of handling instruments and carrying out patient examinations [26,27,28]. Several of these techniques can be adopted in the optometry context to learn and refine eye examination skills. ICT approaches can further be used as additional modes to improve skills in ocular electrophysiology and image analysis as well as evaluation and assessment of students.

Online learning activities are completely internet based and the web based delivery forms the basis of delivering the learning material and assessing the students. Web accessibility is therefore critical for the successful learning and teaching activities. Students in most of the developing and developed countries have reasonable access to high-speed internet in optometry schools and clinical environments [29,30]. Educational tasks can make use of large numbers of online servers and several applications such as bioinformatics data bases, image analysis software, wiki resources, biostatistics and open access articles to bring a positive influence towards the learning of optometry students and their professional development. Several resources such as cloud storage of materials (dropbox), e-portfolio (Linkedln etc.), open education resources such as creative commons, pubmed, google scholar etc., and Echo 360, skype, screenFlow for lectures online are being increasingly used. In addition, teachers can make use of chat rooms and google hangouts for webinars and lectures [31]. Other interactive platforms such as use of blogs, discussion forums, group emails and messages, journals, google drives as well as social media such as facebook, twitter and instagram have immense potential to increase student engagement and for learning and teaching objectives [32]. New forms of learning involving hypertexts, hypermedia, interactive 3D-simulations, video-conferences, educational blogs, discussion platforms and webinars may be stimulating experiences for optometry students. Remote log-in software such as Team Viewer have immense educational value where students and teachers can remotely log-in to the computers from any place in the world. This can be used to assess files and software applications from home or during travel and may be particularly relevant for optometrists and ophthalmologists working in rural settings [33]. For assessment of students, again ICT is helpful in designing and following rubrics, improving formal and self-assessment by using turnitin and quizzes and improving access to data bases relevant to vision sciences and optometry by using public and institutional wiki resources [34]. Unit specific specialized optometry content can be delivered in an explicit and much more interactive manner when compared to conventional teaching involving text, diagrams and images from case studies. Appropriate supervision however is necessary to keep the learning plan focused to ensure best time management and evade potential cognitive overload. While computer-based learning trains independence and responsibility, moderation by trained personnel is necessary to align the dialogue between students and teachers as well as the study approach with the relevant learning objectives in the optometry units [35]. It is equally important that students and professionals using discussion forums, group emails, social media, google drives and clouds, patient data transmission software and storage servers are well-aware of the information governance and storage regulations, intellectual property and ethical issues in the context of education and patient confidentiality [36,37].

Virtual reality techniques can help generate an artificial ambiance and facilitate optometry students’ interface with the external environment. To enhance learning interface and student interest, the information can be visualized as 3D correlative disposition [38,39]. Virtual reality techniques are immensely useful in understanding bioinformatics applicability and can improve learning outcomes in pharmacology, anatomy, biochemistry and therapeutics units of optometry and allied sciences. Virtual reality can also have immense applications in health informatics, for example in using various OCT and retinal scanning tools and data storage. These can also be used in MRI data mining and to delineate correlations of brain findings with vision changes using complex and custom made analytical software. A particularly useful aspect is the ability of the virtual environment to support multiple users simultaneously which can be used to promote interactive and collaborative learning in optometry and other health sciences [40,41,42]. Wider usage of virtual reality techniques may help to enhance the learners’ initiatives to study as compared to a purely teacher initiated learning. Recent advances in mobile and tablet technology and their community wide popularity has opened several perspectives to use these tools as learning mediums to deliver educational links and materials for improved outcomes. In addition to providing learning alternatives, familiarity with these technologies can help the professional optometrist to examine the patients in remote areas and outside the clinic environment settings [43]. For example, several applications are being developed on tablets and smart phones to assess the visual fields, pupil response and diagnose vision defects which may have useful applications in different scenarios including in rural places [44].

Together, these learning aids provide excellent means to improve traditional face-to-face teaching and have the potential to improve optometry, ophthalmology and orthoptist professional health care capabilities. These approaches can be effectively used to design the curriculum for various levels and provide constructive assessment to students. These will also be useful for continuing professional development for registered optometrists and allied professionals, including ophthalmologists, orthoptists and vision scientists. ICT advances and the increasing availability of information in the public domain on various aspects of assessment and management of vision problems is helpful in disseminating awareness on eye checks and eye diseases for patients/carers and professionals [45].

## 4. Factors Impeding and Facilitating Implementation of ICT Learning

A major barrier in the wider implementation of ICTs in optometry classroom and clinical education is the shortage of ICT trained teachers and this is generally true for most of the health care disciplines [46]. Most optometry teachers have a background in vision sciences, optometry or related disciplines and are not specialists in implementing digital education, bioinformatics, health informatics tools and software. Recruiting lecturers qualified in utilizing ICT technologies will help in streamlining the use of e-learning technologies in optometry. A clear health education policy along with institutional planning, execution and support from top management is necessary to implement greater use of technology in learning [47]. Increased collaboration of optometry disciplines with centers of excellence in bioinformatics, ICT learning technologies and health informatics will impart further depth to the adaptation of optometry courses to changing times with much greater emphasis on e-learning and blended teaching [21,48].

Another major factor is that most academic staff in optometry schools have clinical responsibilities and as such can spend only limited time in learning innovative technologies and adapting them in teaching practice. Teachers’ confidence in the effectiveness of ICTs in education and their technological know-how can potentially be improved by imparting regular training in workshops and short-courses. The motivation of instructors to attend these training and development courses can be increased by adapting a flexible training schedule and linking rewards with attendance as well as greater co-ordination from the administration. These ICT training sessions can be designed in the context of optometry specific requirements and challenges which will also help to refine the clinical techniques of practicing optometrists, in addition to improving teaching practice, social engagement and knowledge update [49].

Optometry students, particularly in the undergraduate courses, may also experience initial hesitation in adapting to the use of ICTs in studies and face problems until a comfortable level of expertise is achieved in using technologies and software. These problems may range from basic trouble-shooting such as difficulties in accessing the online learning materials, thereby making the whole process time-consuming to delayed responses from the unit conveners. Occasionally, these hurdles are compounded by a lack of systematic and comprehensive educational content through online access. It is to be emphasized that, if the difficulties persist for a long time and are not addressed promptly, many students are likely to lose interest and explore more conventional ways within a familiar comfort zone. Thus, it is important for the teachers and coordinators to ensure their materials are easily accessible to students regardless of the time and place [50]. Greater use of visual and sound effects can help improve the motivation, interest and engagement of optometry students. Peer support and collaborative learning through blended approaches, specifically in the optometry context, can similarly help to attract and retain students and these approaches become more important in the online space [51].

ICT mediated approaches using virtual patients are being increasingly used to study and train in many health training programs and greater use of this approach will allow more accessibility and cost-effective case-based learning in optometry [52,53]. This will also allow students to carry out clinical studies at a time of their own convenience. This clinical learning experience combined with virtual environments using ICTs will increase student engagement and provide diverse educational exposure with better learning outcomes. Students with limited skills in ICTs and different educational backgrounds may face difficulties, but in the computer and mobile phone era it is increasingly hard for the curriculums to ignore the in silico learning and teaching dimension without the threat of becoming outdated and obsolete.

A major benefit of implementing ICT mediated educational engagement is radically improved communication between the students and teachers. It helps the teachers and students to acquire and pass teaching notes and engage with peers for social construction of knowledge and building analytical capabilities. It is however important to prepare high quality customized educational material, suitable for digital delivery of optometry content and to make the material more attractive and user friendly. Blended learning for optometry students and for the purpose of continuing professional development facilitates flexible learning where students can determine the pace of their own study while still following the course schedule [54].

For complex bioinformatics and health informatics related software, a pre-requisite is high-speed processers for the computers and good quality internet connection. This is required for real-time modelling, webinars, 3D image processing and virtual reality programmes. Insufficient processing power of computers and low-speed internet connections can be a great hindrance in retaining students for ICT mediated learning approaches. The advent of high-speed fiber-optic technology and advanced computers has largely overcome the technical issues and it is primarily up to the institutional managements to invest judiciously and acquire appropriate learning in silico platforms and software to provide the best possible learning and teaching technologies to optometry students and teachers for the best learning experiences and outcomes [55].

## 5. Prospects in Various Stages of Optometry Education

Use of ICT technologies in optometry education has great potential in complementing existing programs to enrich the learning experiences of students and teachers and enhance learning outcomes. ICTs are increasingly playing a central role in blended learning and e-learning approaches which can be integrated into optometry education to make it suitable for learners from different backgrounds and to increase the attractiveness of the optometry programs. Several schools of optometry now incorporate a large aspect of online learning in addition to conventional classroom lecturing [20]. While basic ICT tools and associated e-learning have applications in physics, basic biology, physiology, ocular pathology and anatomy oriented units; bioinformatics and health informatics tools are more relevant to therapeutics and clinical optometry units and in the research projects. ICTs may be used to provide explicit instructions to students about the cognition of drugs, safe drug handling and novel therapeutic and diagnostic methods and professional ethics [56]. The contribution of technology to learning can be evaluated by comparing student engagement and evaluating responses to specific vision related questions. Students’ perceptions can be obtained by assessment questionnaires using online quizzes. Lectures, discussions and students’ responses can be recorded for evaluation and compliance. It is generally observed that students are more interested in audio-visual and digital approaches compared to conventional classroom and lab based teaching and the learning impact is long-lasting. The effects can be determined by recording the time spent by students on these tools and correlating it with students’ grades. Judicious use of ICTs helps students to understand the concepts rather than purely memorizing, and develops more effective analytical and critical thinking abilities which have applications in optometry [57]. These outcomes are in alignment with the higher education and training objectives to promote long-lasting education and continuing professional development. Like most medical practitioners, qualified optometrists are more likely to take part in online educational programs due to work commitments and wider the implementation of ICT will help to implement long-term continuing education plans for optometry practitioners to acquire competency and professional accreditation [58].

A combination of audio-visual material with e-books, notes and extensive use of the internet and computers can open up exciting new avenues for e-learning opportunities in optometry to meet educational needs of students with different backgrounds. One of the technologies that facilitates the advances in e-learning is audio and video streaming [59]. Using this technology, lecturers are able to record videos and deliver teaching materials to the students online with the help of the internet, such as i-tutorials [60]. The method will be beneficial for optometry students as the lecturers can explain the subject step-by-step and in more interactive ways [61]. Data, files and assignments can be easily shared through file sharing technologies such as dropbox, icloud and google drive etc. Several of these can be used to study the material on mobile phones and tablets. Mobile learning facilitates easy access of internet in various places such as during travel, and studies show that most students welcome increased engagement with mobile learning tools [62]. Smart phones and other mobile technology has allowed students to make constructive use of travel and leisure time for educational purposes. This also allows better usage of resources such as lecture halls etc. ICTs, along with blended learning, facilitate student engagement independent of space and time; the speed of learning can also be individually adapted to the previous knowledge and special needs of optometry students at various levels. This also allows lecturers to engage and communicate with the students outside the lecture time [2]. In this regard, while mobile learning is helpful, it is difficult to implement as an exclusive delivery approach as mobile internet tariff plans can vary greatly and students with different providers may have different tariff rates for the internet usage which is generally a limiting factor [63]. However, building on these technologies that have been greatly useful in several other health care disciplines can greatly benefit the optometry and allied health care students and professionals.

The rapid growth of technologies and wide internet availability has encouraged many universities to start incorporating ICTs to enhance e-learning and blended learning. Universities are increasingly recruiting qualified lecturers with experience in e-teaching and blended learning technologies. A rather ignored aspect is that the advent of these newer technologies also facilitates learning among the deaf, hearing-impaired or other physically challenged students and the audio-visual materials can be modified to enhance the use of sign language and subtitles to make the studies more inclusive and help the students learn independently [64]. As more and more international students with diverse cultural and language backgrounds take to optometry, orthoptics and ophthalmology training, especially in developed countries, ICTs and associated blended learning technologies hold the promise to enhance their participation in the discussions and educational activities by encouraging them to participate in collaborative activities with peers [65]. Virtual communication facilitated by ICTs helps in decreasing existing or perceived gender or ethnic inequalities that may occur in the face-to-face communication. For example, female students may feel more sense of community and be conscious of the presence of colleagues compared to male students and cause differences in communicative styles during their learning activities. However, some gender differences are also observed with digital learning where female students are generally more active on social networking tools and online discussions, whereas males may be more oriented towards the technological aspects of the tools. However, most studies indicate that there are no significant gender differences in the awareness of e-learning implementation and overall effects of gender differences in digital learning may not be as prominent as in conventional classroom learning [66]. Greater use of ICTs to facilitate blended learning in optometry will help narrow the gap and provide alternative platforms to groups of students who may feel disadvantaged in the classroom teaching.

ICTs facilitate collaborative learning by enhancing opportunities for blended learning, stand-alone e-learning and also through synchronous virtual classroom technology supplemented by group-based learning for better educational outcomes. Students can be educated about various technologies that are effective in improving the clinical practice, such as reminder techniques for patients, including e-mails, digital awareness posters, flyers and messaging services [67]. The wider use of technology for educational and professional purposes will improve the curriculum by widening the scope of diagnostic and prognostic challenges, using virtual microscopy and digital pathology components [68].

## 6. Students’ and Teachers’ Perception

With the increasing use of ICTs in university health care education and its increasing adaptation in optometry courses, the need to understand the students’ and teachers’ perception on this matter has become vital to understanding discipline specific relevance and acceptability. It is important to understand the students’ and teachers’ acceptance of using ICT as part of their learning modes and its benefits, so that appropriate changes and improvements can be made by policy-makers and professional bodies. Various studies show acceptance of ICT and e-learning technologies among students as moderate with some concerns having been raised about the unfamiliar and rapidly evolving in silico environments associated with most of these techniques. These concerns however vary with students’ background and can be addressed, to a great extent, with lecturers’ and institutional support. Other factors, such as relevance of material to unit content, appropriate software selection and forming suitable discussion and learning groups can improve ICT acceptability and their outcomes in learning [50]. Further, the use of technology and discipline specific e-learning applications may be more relevant in postgraduate compared to undergraduate courses as many professional optometrists with experience of using technology in practice may be more accepting of the use of ICT applications and online learning. Besides, it may be easier to “design and sell” online programmes for postgraduate and higher degree research students who are already acquainted with several of the instrumentation and practice related ICT applications and generally have a higher motivation to complete courses due to professional and family commitments.

An educational improvement effect or otherwise can be assessed by evaluating knowledge gained, confidence, interest, and participation in online and subsequent classroom discussions. Studies show that the majority of students are open to ICT use in an educational approach as long as the contents are well organized and easy to understand [69]. Besides, ICTs open up a whole new sphere of online forums and threads for students and they can engage with teachers and peers at a time and place of their choice. This way, students get greater opportunity to lead their own discussions, to share knowledge among peers, and get sufficient time to reconstruct their thoughts and prepare their write ups in a much clearer way. These characteristics are especially useful for the international students with limited English fluency to express their thoughts and ideas. Student acceptance of digital learning can be monitored by how much time they spend in forums and the quality of their postings [70].

It is important that the lecturers’ opinions on newer technology are taken into consideration as their familiarity with digital learning and their experiences are critical to implement newer ICTs. However, teachers need to be trained and exposed to ICT policy and implementation as well. This can be achieved through training, guidebooks and induction programs as well as by attaching certain incentives, gradually progressing towards using particular unit-specific ICT approaches [21]. Most of the institutions however do not compensate the teachers for the additional time they spend in learning and adapting to new ICTs—an important factor that management needs to consider for optimal engagement and the best learning outcomes. Students’ and teachers’ efforts need to be appropriately supported by faculty, departments, institutional management and professional bodies [71].

## 7. Conclusions

ICTs represent an emerging and promising way to supplement conventional learning in undergraduate and postgraduate programs as well as in professional developments in optometry. Although there are several perceived benefits, a major issue is the assessment of the success of ICTs in educational outcomes in the short and long-term. In the short-term, ICTs can be examined online or in classrooms and clinics in terms of engagement or concept retention and performance in clinical or classroom tests, but for the longer-term, assessments that are more suitable modes of evaluation need to be developed. To delineate the effects, closer collaboration between educational professionals on one side and optometrist, orthoptists, vision researchers and ophthalmologists on the other side is required. Development of sustainable concepts will help in progressive engagement of ever-expanding vision science horizons while striking a balance between students’ expectations and performance with institutional objectives and resources. It needs to be determined how ICTs improve the equilibrium between employment prospects and specific educational and scientific priorities. Extensive use of ICTs in the optometry curriculum will be sustainable in an everyday academic routine if its additional value as compared to traditional forms of teaching and learning is obvious. Greater awareness of ethical aspects, such as digital handling of the patient data and clinical trials will become increasingly important with the growing use of ICTs. A close collaboration between information technology professionals, teachers, trained optometrists, professional bodies and institutional management is necessary to generate ambitious programs in optometry and other allied vision fields in the years to come.
